# Impact of neuroradiologist second opinion on staging and management of head and neck cancer

**DOI:** 10.1186/1916-0216-42-39

**Published:** 2013-06-05

**Authors:** John T Lysack, Monica Hoy, Mark E Hudon, Steven C Nakoneshny, Shamir P Chandarana, T Wayne Matthews, Joseph C Dort

**Affiliations:** 1 Division of Neuroradiology, University of Calgary, Calgary, Alberta, Canada; 2 Division of Otolaryngology-Head and Neck Surgery, University of Calgary, Calgary, Alberta, Canada; 3 The Ohlson Research Initiative, University of Calgary, Calgary, Alberta, Canada; 4 Department of Diagnostic Imaging, Foothills Medical Centre, 1403-29 Street NW, Calgary, AB T2N 2T9, Canada

**Keywords:** Second opinion, Radiology, Cancer staging, Head and neck cancer, Quality improvement

## Abstract

**Objective:**

Patients with head and neck cancer frequently present to academic tertiary referral centers with imaging studies that have been performed and interpreted elsewhere. At our institution, these outside head and neck imaging studies undergo formal second opinion reporting by a fellowship-trained academic neuroradiologist with expertise in head and neck imaging. The purpose of this study was to determine the impact of this practice on cancer staging and patient management.

**Methods:**

Our institutional review board approved the retrospective review of randomized original and second opinion reports for 94 consecutive cases of biopsy proven or clinically suspected head and neck cancer in calendar year 2010. Discrepancy rates for staging and recommended patient management were calculated and, for the 32% (30/94) of cases that subsequently went to surgery, the accuracies of the reports were determined relative to the pathologic staging gold standard.

**Results:**

Following neuroradiologist second opinion review, the cancer stage changed in 56% (53/94) of cases and the recommended management changed in 38% (36/94) of patients with head and neck cancer. When compared to the pathologic staging gold standard, the second opinion was correct 93% (28/30) of the time.

**Conclusion:**

In a majority of patients with head and neck cancer, neuroradiologist second opinion review of their outside imaging studies resulted in an accurate change in their cancer stage and this frequently led to a change in their management plan.

## Introduction

Despite advances in diagnosis and treatment, head and neck cancer remains an important cause of death and disability worldwide. The most recent global estimates from the World Health Organization indicate that 845,000 new cases of head and neck cancer are diagnosed each year, with a mortality rate approaching 50% [1].

Head and neck cancer may present with a variety of symptoms and signs depending, in part, on the site of the primary tumor. The diagnosis is usually made or suspected on the basis of the clinical examination and confirmed with a biopsy of the primary lesion or a metastatic lymph node. The most important prognostic factor, after primary site, tumor type, and tumor grade, is cancer stage and, all else being equal, high-stage cancers have a poorer prognosis than low-stage cancers. Cancer stage is also a major consideration when determining appropriate treatment. For example, for some primaries, the recommended management is surgical for low-stage cancers and nonsurgical (e.g. chemoradiotherapy) for high-stage cancers. Because the primary site is usually identified clinically, and the tumor type and grade are determined by pathology, the main role of imaging in the initial management of patients with head and neck cancer is to facilitate accurate staging.

At academic tertiary referral centers, patients frequently present with imaging studies that have been performed and interpreted elsewhere [2]. At our institution, all outside head and neck imaging studies undergo formal reinterpretation by a fellowship-trained academic neuroradiologist with expertise in head and neck imaging prior to discussion of the case at a weekly multidisciplinary tumor board. At the weekly tumor board, the multidisciplinary team reviews the clinical, pathological, and imaging data, the cancer stage is determined, and a treatment recommendation is made. The purpose of this study was to determine how frequently the neuroradiology second opinion changed staging and management, and to estimate how frequently the second opinion was accurate when compared with surgical findings.

## Methods

Our institutional review board (The University of Calgary Conjoint Health Research Ethics Board) approved the retrospective review of patient data for this study, and waived the requirement for informed consent. All cases (n = 94) of biopsy proven or clinically suspected head and neck cancer presenting to our academic tertiary referral center with outside computed tomography (CT) or magnetic resonance (MR) imaging during the 2010 calendar year were retrospectively identified from our institutional head and neck cancer database (Table 1). As part of our routine practice, a fellowship-trained academic neuroradiologist with expertise in head and neck imaging and three years of post-fellowship experience had issued a formal second opinion report in all cases. Hardcopies of the original and second opinion reports were collected, randomized, and distributed to three reviewers: a fellowship-trained academic head and neck surgeon with 20 years of post-fellowship experience, an otolaryngology-head and neck surgery resident with four years of residency experience, and a fellowship-trained academic neuroradiologist with 15 years of post-fellowship experience (a different neuroradiologist than the one who had issued the second opinion reports).

**Table 1:** Patient and tumor characteristics

	**All cases**	**Surgical cases**
**Variable**	**(n = 94)**	**(n = 30)**
Age (y), mean (range)	61.6 (31.1, 90.1)	65.2 (36.3, 89.7)
Gender (M:F)	70:24	17:13
Primary		
Oral Cavity	24 (25.5%)	19 (63.3%)
Oropharynx	22 (23.4%)	2 (6.7%)
Hypopharynx	2 (2.1%)	0 (0.0%)
Larynx	15 (16.0%)	3 (10.0%)
Salivary Gland	6 (6.4%)	3 (10.0%)
NC & PNS	5 (5.3%)	2 (6.7%)
Nasopharynx	7 (7.4%)	0 (0.0%)
Thyroid	1 (1.1%)	0 (0.0%)
Unknown	5 (5.3%)	0 (0.0%)
Skin	4 (4.3%)	1 (3.3%)
Lymphoma	3 (3.2%)	0 (0.0%)
Stage		
Benign	2 (2.1%)	1 (3.3%)
Stage 0	3 (3.2%)	2 (6.7%)
Stage I	9 (9.6%)	4 (13.3%)
Stage II	3 (3.2%)	2 (6.7%)
Stage III	9 (9.6%)	3 (10.0%)
Stage IV	68 (72.3%)	18 (60.0%)

NC & PNS, Nasal Cavity and Paranasal Sinuses.

The three reviewers independently analyzed each of the 188 randomized reports (94 original radiology reports and 94 second opinion reports) and recorded the primary site, T-category (extent of primary tumor), and N-category (extent of cervical lymph node metastases) using the most recent American Joint Committee on Cancer definitions [3], as could be determined from the information found in each radiology report. To determine the reliability of the data, the inter-rater agreements for T-category and N-category for the original and second opinion reports were calculated using Fleiss' κ for multiple raters [4]; the opinion of the head and neck surgeon was considered the gold standard.

The cancer stage (‘Stage 0-IV’) was calculated from the T-category and N-category data according to the most recent American Joint Committee on Cancer definitions [3], as all patients had no evidence of distant metastases. Staging data were then dichotomized into low-stage (Stage 0, I, or II) vs. high-stage (Stage III or IV) groups (‘Low/High Stage’), and into node-negative (N0) vs. node-positive (N1, N2, or N3) groups (‘Node Negative/Positive’). The staging data derived from the original reports were compared with those from the second opinion reports, and discrepancy rates with 95% confidence intervals (95% CI) were calculated using the modified Wald method [5]. If there was a change in staging from the original report to the second opinion, the direction of change (up-staging vs. down-staging) was determined.

The head and neck surgeon also made a recommendation for patient management based on the information provided in each radiology report. He was asked, “Assuming that the information in the radiology report is correct and agrees with your clinical impression, and that the patient is agreeable, otherwise healthy, and has no contraindications, what initial management would you recommend?” His choices were: (a) surgery (resection of the primary tumor +/−lymph node dissection); (b) radiotherapy (+/−chemotherapy); (c) no treatment; and (d) don't know. For the ‘don’t know’ cases, the surgeon recorded the reason. The surgeon’s management recommendations based on the original reports were compared with those based on the second opinion reports, and discrepancy rates with 95% confidence intervals (95% CI) were calculated using the modified Wald method [5]. Fisher’s exact test [6] was used to reject the null hypothesis that the surgeon’s management recommendations based on the original and second opinion reports were the same.

For the subgroup of cases (n = 30) in which surgery was subsequently performed, pathologic staging data were acquired from pathology reports. The radiologic staging data were compared to the pathologic staging data and the accuracies of the original and second opinion reports were calculated. Fisher’s exact test [6] was used to reject the null hypothesis that the accuracies for cancer stage of the original and second opinion reports were the same.

All tests were two-sided tests using a threshold for statistical significance of *P* < .05. Statistical analysis was performed using Stata 11 (StataCorp, College Station, TX).

## Results

The staging data that were extracted from the radiology reports were found to be reliable. There was ‘moderate’ to ‘substantial’ inter-rater agreement for the original reports (κ = 0.44 for T-category and κ = 0.78 for N-category) and ‘substantial’ to ‘almost perfect’ agreement for the second opinion reports (κ = 0.62 for T-category and κ = 0.89 for N-category) [7].

The discrepancy rate for cancer stage (Stage 0-IV) was 56.4% (95% CI, 46.3% − 65.9%); the discrepancy rates for staging ranged from a low of 34.0% (95% CI, 25.2% − 44.1%) for both Low/High Stage and Node Negative/Positive to a high of 66.0% (95% CI, 55.9% − 74.8%) for T-category (Table 2). Up-staging was common, occurring in 90.6% (48/53; 95% CI, 79.3% − 96.3%) of discrepant cases.

**Table 2:** Discrepancies in cancer staging between the original and second opinion radiology reports

	**All cases**	**Surgical cases**
**Staging criteria**	**(n = 94)**	**(n = 30)**
T-category	62 (66.0%)	18 (60.0%)
N-category	50 (53.2%)	13 (43.3%)
Stage (0-IV)	53 (56.4%)	17 (56.7%)
Low/High Stage	32 (34.0%)	11 (36.7%)
Node Negative/Positive	32 (34.0%)	9 (30.0%)

**Figure 1:**
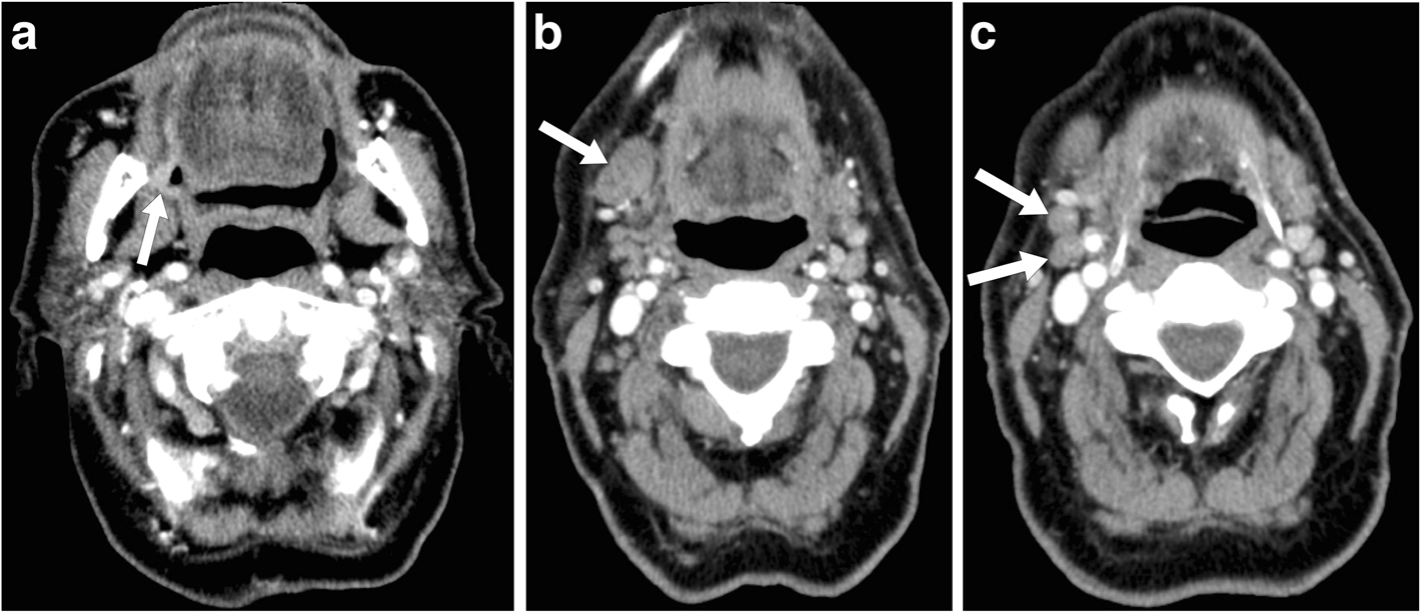
**Axial contrast enhanced CT images of the neck in an 81 year old male (Patient Z in Table** 3**); a fine needle aspiration biopsy of a clinically enlarged right submandibular lymph node had shown squamous cell carcinoma. (a)** A plaque-like (2.5 cm in diameter × 0.5 cm in maximum thickness) enhancing mucosal lesion involving the right retromolar trigone (arrow) was not mentioned in the original radiology report but was identified as the primary tumor in the second opinion report. **(b)** An enlarged (2.3 cm × 1.5 cm) right level IB lymph node (arrow) was interpreted as metastatic in both the original and second opinion reports. **(c)** Two sub-centimeter but rounded and asymmetrically prominent right level IIA lymph nodes (arrows) were not mentioned in the original report but were interpreted as metastatic in the second opinion report. Staging was TxN1 based on the original report and T2N2b (Oral Cavity) based on the second opinion report, and the recommended management was ‘don’t know’ based on the original report (because the primary tumor location and T-category were unclear) and ‘surgery’ based on the second opinion report. Surgery was subsequently performed and the final pathologic staging was T2N2b (Oral Cavity).

Pathologic staging data were available for the 30/94 (31.9%) of cases in which surgery was subsequently performed (Table 3). When compared to the entire group, the surgical subgroup had a higher proportion of oral cavity primaries and a lower proportion of oropharyngeal primaries (Table 1), which reflects the fact that oral cavity cancers are usually treated surgically while oropharyngeal cancers are usually treated with radiotherapy +/−chemotherapy at our institution. In the surgical cases, the original radiology reports agreed with the pathologic stage (Stage 0-IV) 40.0% (12/30) of the time (Table 4) whereas the second opinion reports agreed with the pathologic stage 93.3% (28/30) of the time (*P* < .001). Following the second opinion, the cancer stage changed in 56.7% (17/30) of the surgical cases, and these changes were pathologically proven to be correct 94.1% (16/17) of the time (Figure 1).

**Table 3:** Cancer staging and recommended management based on the original and se1cond opinion radiology reports compared to the pathologic staging gold standard (n = 30)

					**Staging**			**Management**	
**Pt**	**Age**	**M/F**	**Primary**	**Original**	**Second opinion**	**Pathologic**	**Original**	**Second opinion**	**Actual**
A	89	F	Oral Cavity	TxN0	T4aN2b	T4aN0	Don’t know^†^	Surgery	Surgery
B	55	M	Oral Cavity	T0N0	T1N1	T2N1	No treatment	Surgery	Surgery
C	84	F	Oral Cavity	T2N0	T2N0	T2N2b	Surgery	Surgery	Surgery
D	40	M	NC & PNS	T0N0	T3Nx	T3Nx	No treatment	Don’t know^‡^	Surgery
E	51	F	Oral Cavity	TxN0	TxN0	TisN0	Surgery^*^	Surgery^*^	Surgery
F	36	M	Oral Cavity	TxNx	T4aN0	T4aN0	Don’t know^†^	Don’t know^‡^	Surgery
G	68	M	Larynx	TxNx	T1N0	T1N0	Don’t know^†^	Surgery	Surgery
H	75	F	Oral Cavity	T1N2b	T1N2b	T1N2c	Surgery	Surgery	Surgery
I	74	M	Salivary Gland	T2N0	T2N0	T2N0	Surgery	Surgery	Surgery
J	69	M	Larynx	T4aN1	T4aN2b	T4aN1	Surgery	Surgery	Surgery
K	61	M	Oral Cavity	TxN0	T2N0	T2N0	Don’t know^†^	Surgery	Surgery
L	80	F	Oral Cavity	T2N0	T1N1	T2N2b	Surgery	Surgery	Surgery
M	54	M	Oropharynx	TxN1	T1N1	T1N1	RT^§^	RT^§^	Surgery
N	82	F	Oral Cavity	T0N0	T0N0	T0N0	Don’t know^‡^	Don’t know^‡^	Surgery
O	49	F	Oral Cavity	T0N0	T1N0	T1N0	No treatment	Don’t know^‡^	Surgery
P	70	M	Larynx	T4aN0	T4aN2c	T4aN0	Surgery	Surgery	Surgery
Q	90	M	Oral Cavity	TxN1	T2N1	T2N1	Don’t know^†^	Don’t know^‡^	Surgery
R	85	F	NC & PNS	TxN0	T4aN0	T4aN0	Don’t know^†^	Don’t know^◊^	Surgery
S	56	M	Oral Cavity	TxN0	T4aN0	T4aN2b	Surgery^*^	Surgery	Surgery
T	58	M	Oral Cavity	T4aNx	T4aN2c	T4aN1	Don’t know^†^	Surgery	Surgery
U	45	M	Salivary Gland	T1N0	T1N0	T1N0	Surgery	Surgery	Surgery
V	88	F	Oral Cavity	TxN1	T4aN1	T4aN1	Don’t know^†^	Surgery	Surgery
W	56	F	Oral Cavity	T0N0	TxN0	TisN0	No treatment	Don’t know^‡^	Surgery
X	43	M	Oropharynx	TxN2a	T2N2b	T2N2b	Don’t know^†^	RT^§^	Surgery
Y	50	F	Oral Cavity	T1N0	T1N0	T1N0	Don’t know^‡^	Don’t know^‡^	Surgery
Z	81	M	Oral Cavity	TxN1	T2N2b	T2N2b	Don’t know^†^	Surgery	Surgery
AA	81	M	Skin	TxN2b	TxN2b	TxN2b	Don’t know^◊^	Don’t know^◊^	Surgery
AB	58	F	Oral Cavity	T2N2b	T2N2b	T2N2b	Don’t know^‡^	Don’t know^‡^	Surgery
AC	63	F	Salivary Gland	T3N0	T4aN2b	T4aN1	Surgery	Surgery	Surgery
AD	65	M	Oral Cavity	TxN1	T2N2b	T2N2b	Don’t know^†^	Surgery	Surgery

F, Female; M, Male; NC & PNS, Nasal Cavity and Paranasal Sinuses; Pt, Patient Identifier; RT, Radiotherapy (+/−chemotherapy).

^*^ Biopsy proven oral cavity cancer was stated in the Clinical History section of the report.

^†^ Insufficient information in the report to make a recommendation.

^‡^ Technically inadequate or incomplete scan; the radiologist recommended additional imaging.

^§^ Oropharyngeal carcinoma is usually treated with primary radiotherapy (+/−chemotherapy) at our institution.

^◊^ Post-treatment recurrence; the surgeon needed details of prior treatment before making a salvage treatment decision.

**Table 4:** Accuracies of the original and second opinion radiology reports compared to the pathologic staging gold standard (n = 30)

**Staging criteria**	**Original report**	**Second opinion**
T-category	43.3% (27.4% − 60.8%)	93.3% (77.6% − 99.2%)
N-category	56.7% (39.2% − 72.6%)	70.0% (52.0% − 83.5%)
Stage (0-IV)	40.0% (24.6% − 57.7%)	93.3% (77.6% − 99.2%)
Low/High Stage	60.0% (42.3% − 75.4%)	96.7% (81.9% − 99.9%)
Node Negative/Positive	70.0% (52.0% − 83.5%)	86.7% (69.7% − 95.3%)

Data in parentheses are 95% confidence intervals.

The discrepancy rate for patient management was 38.3% (36/94; 95% CI, 29.1% − 48.4%); most changes (77.8%; 28/36) were from ‘don’t know’ based on the original report to one of surgery or radiotherapy (+/−chemotherapy) based on the second opinion report (Table 5). It was not possible for the surgeon to make a management recommendation based on either the original report or the second opinion report in 29.8% (28/94) of cases. In most of these cases (75.0%; 21/28), this was because of a technically inadequate or incomplete scan for which the radiologist recommended additional imaging, or a post-treatment recurrence for which the surgeon needed details of prior treatment before making a treatment decision. Despite this, the management recommendation based on the second opinion reports agreed with the actual management 60.0% (18/30; 95% CI, 42.3% − 75.4%) of the time, as compared to 33.3% (10/30; 95% CI, 19.1% − 51.3%) of the time for the original reports (*P* = .069).

**Table 5:** Differences in recommended management based on the original and second opinion radiology reports

**Recommended management**			**Based on second opinion report**		
		**Surgery**	**RT**	**No treatment**	**Don’t know**
	Surgery	14 (14.9%)	1 (1.1%)	0 (0.0%)	1 (1.1%)
**Based on original report**	RT	0 (0.0%)	15 (16.0%)	0 (0.0%)	1 (1.1%)
	No treatment	1 (1.1%)	1 (1.1%)	1 (1.1%)	2 (2.1%)
	Don’t know	16 (17.0%)^*^	12 (12.8%)^†^	1 (1.1%)	28 (29.8%)

RT, Radiotherapy (+/−chemotherapy).

^*^P < .001.

^†^*P* = .002.

## Discussion

Our study demonstrates the value of reinterpretation of head and neck imaging studies by a fellowship-trained academic neuroradiologist; such reinterpretation led to more accurate staging and better treatment decision-making. We found that, following formal second opinion reporting of outside imaging studies, there was a change in cancer stage in 56% of cases, resulting in a change in management in 38% of patients; for the surgical cases, the second opinions were pathologically proven to be correct 93% of the time.

These findings add to the evidence that subspecialty radiologist interpretation of imaging studies has a positive impact on patient care. In the head and neck cancer domain, this was first reported by Loevner et al [8]; the authors found a change in cancer stage in 34% of cases and a change in management in 40% of patients following expert radiologist reinterpretation. More recently, Wheless et al [9] showed that, following case review at a multidisciplinary head and neck tumor board, 27% of patients had a change in tumor diagnosis, stage, or treatment plan. In other oncological and non-oncological domains, studies of radiologist second opinions have found discrepancy rates of 11–49% for diagnosis or staging and 7–37% for patient management [10-16]. These rates are similar to those found for expert second opinions in pathology, with reported discrepancy rates of 7–66% (including changes from a benign to a malignant diagnosis or vice versa) resulting in a change in patient management in 1–28% [17-28]. There is much less literature on the effect of expert second opinions in clinical practice, but one study [29] has shown discrepancy rates for diagnosis and patient management of 35% and 67%, respectively.

New insights into differences in biological behaviors of head and neck cancers are leading to personalized treatment options that are becoming increasingly diverse. Parallel to this trend are advances in biologic imaging of head and neck cancer that are used to select the most appropriate treatment regimen for each individual patient [30]. As imaging techniques become more complex, the frequency of clinically significant differences in interpretation are expected to increase. Although a change in diagnosis following subspecialist reinterpretation of an imaging study does not necessarily imply a *correct* change in diagnosis, “second opinion best reflects a measure of diagnostic precision, which is a practical surrogate for diagnostic accuracy, given the difficulties of defining a gold standard” [31]. Thus, despite their costs in terms of increased and often uncompensated workload [2], it is likely that subspecialist second opinions will become increasingly important for high-quality patient care.

In general radiology practice, where there is the usual mixture of normal with abnormal cases, the discrepancy rate is only 3% [32]. However, for patient populations with 100% prevalence of disease, like those served by multidisciplinary tumor boards at academic tertiary referral centers, the discrepancy rate can increase by an order of magnitude [33]. The benefit of a subspecialist model of radiology practice is highlighted by a recent study [34] that showed the discrepancy rate between fellowship-trained academic neuroradiologists was only 2% despite the high prevalence of disease (92%) in that environment. A strength of the present study is that each radiology report was independently analyzed for staging information by three physician reviewers; an experienced fellowship-trained academic head and neck surgeon, a senior otolaryngology-head and neck surgery resident, and an experienced fellowship-trained academic neuroradiologist (a different neuroradiologist than the one who had issued the second opinion reports). The greater inter-rater agreement found for the second opinion reports supports the assertion that imaging reinterpretation by a subspecialist radiologist results in improved diagnostic precision.

The weaknesses of the current study are its retrospective nonblinded design and the absence of a pathologic gold standard in the nonsurgical cases. We chose a retrospective design to decrease the potential bias of secondary gain by the reinterpreting neuroradiologist who, if he knew his second opinion reports were to be subsequently scrutinized in a prospective study, might be motivated to exaggerate differences of opinion. The trade-off was to accept that the type of report (original vs. second opinion) could not be adequately blinded to the reviewers. Even if the name of the reporting radiologists and the location of the study were removed from the reports, it would be clear, based on the content and style of the report, which were second opinions. For example, imaging findings or interpretations from the original radiology reports (both correct and incorrect), as well as clinical information that might only be known at the time of the second opinion, were routinely acknowledged and discussed in the body of the second opinion reports. It was therefore not possible to redact such identifying information without substantially changing the content of the reports. Because of this, it is possible that the reviewers were biased to assign a greater degree of certainty to information contained in reports they knew to be second opinions. This would be expected to be more of a problem for the patient management recommendation component of the study than for the cancer staging component and might, at least in part, explain the relatively high frequency of ‘don’t know’ recommendations for the original reports. However, this bias reflects the reality of clinical practice in which subspecialists may more frequently seek out additional information as is needed to provide a definitive opinion. Arguably, therefore, blinding the reviewers in this way would make the results less generalizable by inaccurately reflecting routine practice. The randomized presentation of the reports to the reviewers partially offsets this bias.

Establishing a gold standard for staging is difficult for many head and neck cancers. Using surgical pathology as the gold standard is problematic, as many patients do not undergo surgery (e.g. some tumors are primarily treated with radiotherapy) and, in those cases where surgery *is* performed, pathologic staging might be incomplete (e.g. local tumor resection but no lymph node dissection), inaccurate (e.g. is the muscle being infiltrated by tumor on this slide an intrinsic or extrinsic muscle of the tongue?), or insufficient (e.g. was there impaired vocal cord mobility or vocal cord fixation?). Even in cases where complete and sufficient pathologic staging information is available, it is only accurate *at the time of surgery* and not *at the time of the scan*. However, an error introduced by a scan-to-surgery delay would apply equally to the original and second opinion reports so, at minimum, the *relative* increase in accuracy found for the second opinion reports in this study is valid.

## Conclusion

More than one-half of patients with head and neck cancer had a change in clinical stage following second opinion review of their outside imaging studies by a fellowship-trained academic neuroradiologist with expertise in head and neck imaging, and this led to a change in management in greater than one-third of patients. In patients with a biopsy proven or clinically suspected malignancy, subspecialty radiologist interpretation of imaging studies has a positive impact on patient care.

## Competing interests

The authors declare that they have no competing interest.

## Authors’ contributions

JL and JD are responsible for study design, data collection, data analysis, and manuscript preparation. MH and MEH are responsible for data collection and manuscript preparation. SN is responsible for statistical analysis and manuscript preparation. SC and TWM are responsible for manuscript preparation. All authors read and approved the final manuscript.
